# Obstructive sleep apnea and objective short sleep duration are independently associated with the risk of serum vitamin D deficiency

**DOI:** 10.1371/journal.pone.0180901

**Published:** 2017-07-07

**Authors:** Ronaldo D. Piovezan, Camila Hirotsu, Marcia C. Feres, Fatima D. Cintra, Monica L. Andersen, Sergio Tufik, Dalva Poyares

**Affiliations:** 1 Department of Psychobiology, Universidade Federal de Sao Paulo, São Paulo, São Paulo, Brazil; 2 Department of Medicine, Universidade Federal de Sao Paulo, São Paulo, São Paulo, Brazil; University of Rome Tor Vergata, ITALY

## Abstract

**Background:**

Studies demonstrate an association between vitamin D (25(OH)D) deficiency and sleep disturbances, such as obstructive sleep apnea (OSA) and short sleep duration. However, to date, no studies have concurrently and objectively evaluated the effect of these factors on 25(OH)D.

**Objectives:**

To evaluate whether OSA and objective short sleep duration are independently associated with reduced 25(OH)D in an adult population sample.

**Methods:**

A cross-sectional study included 657 individuals from the city of Sao Paulo, Brazil, as part of the ERA project. Participants fulfilled questionnaires and underwent clinical evaluation, polysomnography and blood sample collection for 25(OH)D quantification. OSA was classified into three categories (mild, moderate and severe). The risk of 25(OH)D deficiency was considered as levels<30 ng/mL. Short sleep duration was defined as total sleep time<6 hours.

**Results:**

The risk of 25(OH)D deficiency was observed in 59.5% of the sample, affecting more individuals of the female gender, obese, with African American ethnicity, and those that were smokers, sedentary and presented hypertension and diabetes. In the final logistic model adjusted for age, gender, ethnicity, obesity, smoking, hypertension, diabetes, sedentary lifestyle, seasonality and creatinine serum levels, both OSA and short sleep duration showed significant independent associations with the risk of 25(OH)D deficiency (moderate OSA: OR for 25(OH)D<30 = 2.21, 95% CI: 1.35–3.64, p<0.01; severe OSA: OR for 25(OH)D<30 = 1.78, 95% CI: 1.06–3.00, p = 0.03; short sleep duration: OR for 25(OH)D<30 = 1.61, 95% CI: 1.15–2.26, p = 0.01). After a subgroup analysis, similar results were observed only in participants ≥50 years.

**Conclusion:**

OSA and short sleep duration are independently associated with the risk of 25(OH)D deficiency in an adult population. Age-related changes in vitamin D metabolism and the frequency of sleep disorders may be involved in these associations. Future studies exploring whether 25(OH)D levels may modulate OSA and sleep curtailment-related outcomes are needed.

## Introduction

Apart from bone homeostasis, vitamin D (25(OH)D) has been implicated in an increasing number of physiological mechanisms, including sleep. [1] Several studies have identified vitamin D receptors (VDR) in nearly all tissues in the body, including both neuronal and glial cells in the central nervous system. [2] VDR are present in multiple areas of the human brain, including the prefrontal cortex, cingulate gyrus, thalamus, substantia nigra, hippocampus, and also the hypothalamus, a brain area that regulates sleep-wake cycle among other behaviors. [2–4] Moreover, in the last decade, studies reporting associations between sleep disorders and lower levels of 25(OH)D increased consistently. [5], [6]

Particularly, obstructive sleep apnea (OSA) shares relevant risk factors with 25(OH)D deficit, such as age, obesity, hypertension, kidney disease[7], [8] and diabetes. [9], [10] OSA has also been linked to reduced levels of 25(OH)D, as this sleep disorder leads to sleep fragmentation and daytime sleepiness, which might increase the risk of vitamin D deficiency [10–12]. In fact, more recently, Liguori et al. have found that continuous positive airway pressure (CPAP) has an impact on the vitamin D homeostasis of male OSA patients, reinforcing a potential causal relationship in the association between OSA and lower levels of this hormone. [13], [14] Likewise, subjectively measured short sleep duration has also been associated with lower 25(OH)D levels. [15], [16] Additionally, recent evidence suggested a relationship between objective short sleep duration (measured by actigraphy) and 25(OH)D deficit. [17] In a multi-ethnic study, similar results toward reduced actigraphy-measured sleep duration in those with deficient 25(OH)D were reported. [18]

However, taken together with the current evidence, it is still not possible to conclude if there is a confounding or modifying effect of a) reduced sleep duration on the association between OSA and lower 25(OH)D; b) OSA on the relationship between short sleep duration and lower 25(OH)D. Thus, we hypothesized in the current study that both sleep quality and quantity would be able to influence 25(OH)D. Therefore, this study aimed to investigate whether OSA and short sleep duration measured by polysomnography (PSG) are independently associated with the risk of serum 25(OH)D deficiency in an adult sample of Sao Paulo, Brazil.

## Methods

### Data source

The current study is part of the ERA project, an observational study conducted in Sao Paulo, Brazil, in which individuals with and without OSA were cross-sectionally assessed to stratify potential socio-demographic, clinical, laboratory and image data associated with OSA.

### Population

People from both genders, ≥25 years, residents in the city of São Paulo (latitude -*23*.*533773)*, referred to the Sleep Institute (Sao Paulo, Brazil) over a 24-month period, from 2006 to 2007, and who underwent an overnight full PSG study, were invited to participate in the ERA project. Exclusion criteria were: BMI≥40 kg/m^2^, chronic pulmonary disease based on the spirometric classification, New York Heart Association class III or IV heart failure, unstable angina, atrial fibrillation, uncontrolled hypertension, renal disease, recent hospitalization or severe acute conditions, 25(OH)D replacement, CPAP therapy or any previous surgical procedure targeting OSA. The Ethical Committee of the Universidade Federal de Sao Paulo approved this study (registration number 1546/06), which was conducted according to the ethical standards defined in the 1964 Declaration of Helsinki as well as its subsequent amendments. Written informed consent was obtained from all volunteers.

### Measurements

Questionnaires and physical assessments were conducted for the evaluation of socio-demographic and clinical parameters considered as potential confounder variables. Overnight laboratory-based PSG studies were scheduled according to the availability of the volunteers, trying to respect their usual bedtime. Sleep recording was assessed by polysomnographic technologist using a digital system (EMBLA^®^ N7000, Embla Systems Inc., Broomfield, CO, USA). The following physiological tests were performed: electroencephalography (EEG, C3-A2, C4-A1, O1-A2, O2-A1), electrooculography (EOG, EOG-Left-A2, EOG-Right-A1), electromyography (EMG, muscle of the submentonian region, tibialis anterior muscle, and masseter region), electrocardiography (ECG, derivation V1 modified), and airflow detection by both thermocouple and nasal cannula. Respiratory effort was assessed by inductance plethysmography belts, while snoring and body position, percutaneous oxygen saturation (SpO_2_) and pulse rate were evaluated by EMBLA^®^ sensors (Embla Systems Inc., Broomfield, CO, USA). All PSGs were performed and scored by three technicians following guidelines for sleep studies and were reviewed by a sleep medicine physician. All sleep stages were scored according to standardized criteria for investigation of sleep. [19] EEG arousals and leg movements were scored according to the criteria established by the American Academy of Sleep Medicine (AASM) Manual for Scoring Sleep and Associated Events. [20] Apneas were scored and classified following the recommended rule for adults of the AASM Manual, and hypopneas were scored according to the alternative rule. [20]

On the morning following the PSG exam, all participants had a blood sample collected (12 hours of fasting) for quantification of serum levels of total 25(OH)D. After clotting, tubes were centrifuged and kept at −80°C until assayed. We measured serum 25(OH)D using the ARCHITECT 25(OH)D chemiluminescent microparticle immunoassay (Abbott Diagnostics, Wiesbaden, Germany). This method consists of a delayed one-step immunoassay with a six-point calibration for the quantitative measurement of 25(OH)D in a previously described automated instrument system. [21]

### Statistical analysis

Statistical analyses were conducted using Stata 14 (Stata Corp., College Station, Texas, USA). Sample size estimate based on power analysis deriving from previous studies were not carried out as we performed a secondary analysis of the dataset. Descriptive statistics were used to explore the study variables. Serum levels of 25(OH)D below 30 ng/mL were considered as a risk of 25(OH)D deficiency according to the Institute of Medicine of the United States National Academy of Sciences and the US Endocrine Society. [22], [23] Short sleep duration was considered as PSG-measured total sleep time (TST) lower than 6 hours (360 minutes) while OSA diagnosis was based on an apnea-hypopnea index (AHI) as no OSA (AHI< 5); mild OSA (5≤AHI<15), moderate OSA (15≤AHI<30) and severe OSA (AHI≥30). Other PSG-related variables were sleep efficiency, sleep latency, N1, N2, N3, and REM sleep stages, arousal index (AI), and periodic leg movement index (PLMI).

Based on biological, epidemiological and clinical plausibilities, potential confounders or effect modifiers such as demographics, lifestyle, chronic diseases, and laboratory data were considered in the analyses. Sleep-related variables also tested as potential confounders were categorized: sleep efficiency (< 80% TST (reduced), ≥ 80% TST (normal)); sleep latency (≥ 30min (increased), < 30min (normal)); and N1, N2, N3 and REM sleep stages, AI, and PLMI were categorized into quartiles, as a cut-off point for these variables is not clearly established for a wide age range adult population, including older people. [24] Simple logistic regression modeling was applied to assess the individual effects of PSG measured parameters on the risk of 25(OH)D deficiency. [25] Thereafter, a final multivariate logistic regression model was performed, controlling for potential confounders specified *a priori*: age, gender, self-reported ethnicity (African American/not-African American), smoking (current/non-current), obesity status (obese: body mass index (BMI)≥30 kg/m_2_; non-obese: BMI<30kg/m^2^), seasonality (winter/non-winter), sedentarism (yes/no), creatinine levels (mg/dL), hypertension (yes/no), and diabetes (yes/no). The final model included the sleep-related variables that were significant in the univariate models. Moreover, due to the inclusion of both middle-aged and older adults, a subgroup analysis applying the same final model but classifying individuals in two age groups (<50 years and ≥50 years) was performed. Collinearity among the covariates was evaluated. Hosmer-Lemeshow test was used to assess the goodness-of-fit and the c-statistics to evaluate model discrimination. Results were reported as adjusted odds ratios (aOR) and 95% confidence intervals (CI). A p-value<0.05 was considered statistically significant.

## Results

Of the 950 individuals who were screened, 657 were included and 293 presented any exclusion criteria or did not accept to participate in the study. Among the participants, 391 (59.5%) were at risk for serum 25(OH)D deficiency (25(OH)D<30 ng/mL). Mean age was 52 ± 9.1 years (age range: 28–78 years), and 368 (56%) were women. A higher proportion of women (63.6%), African American (59.4%), current smokers (62.4%), and sedentary individuals (59.5%) were at risk for 25(OH)D deficiency. Obesity (59.3%), hypertension (57.7%), and diabetes (55.7%) were also more frequent among participants at risk for 25(OH)D deficiency. Furthermore, participants at risk for 25(OH)D deficiency were assessed more frequently during the winter time (62%) (Table 1).

**Table 1:** Participants characteristics by categories of serum 25(OH)D among 657 individuals participating in the ERA study.

	Serum 25(OH)D categories (ng/mL)		Total Sample
	<30	≥30	
Baseline sample, n (%)*	391 (59.5)	266 (40.5)	657
**Age, y, mean (SD)**	52.2 (9.4)	51.6 (8.8)	52.0 (9.1)
Gender (Female), n (%)*	234 (63.6)	134 (36.4)	368 (56.0)
Race/Ethnicity (African American), n (%)*	41 (59.4)	28 (40.5)	69 (10.5)
Obesity (BMI≥30)**, n (%)*	124 (59.3)	85 (40.7)	209 (31.8)
Current smoking, n (%)*	63 (62.4)	38 (37.6)	93 (14.2)
Hypertension, n (%)*	157 (57.7)	115 (42.3)	272 (41.4)
Diabetes, n (%)*	39 (55.7)	31 (44.3)	70 (10.7)
Sedentarism, n (%)*	238 (59.5)	162 (40.5)	410 (62.4)
Seasonality (winter), n (%)*	98 (62.0)	60 (38.0)	158 (24.1)
**Serum creatinin (mg/dL), mean (SD)**	1.10 (4.6)	0.90 (0.2)	1.02 (3.5)
**Sleep latency (min), mean (SD)**	22.4 (26.3)	21.9 (25.8)	22.2 (26.1)
**Sleep efficiency (%), mean (SD)**	80.1 (14.1)	79.8 (12.1)	80.0 (13.3)
**Arousal index (events/hour), mean (SD)**	17.8 (18.2)	22.7 (16.6)	21.3 (74.4)
Objective short sleep duration (<6h), n (%)*	164 (54.3)	138 (45.7)	302 (46.0)
**Obstructive sleep apnea (OSA) categories**			
*No-OSA*, n (%)*	129 (66.2)	66 (33.9)	195 (29.7)
*Mild*, n (%)*	114 (63.0)	67 (37.0)	181 (27.6)
*Moderate*, n (%)*	67 (50.8)	65 (49.2)	132 (20.1)
*Severe*, n (%)*	81 (54.4)	68 (45.6)	149 (22.7)

* % of total sample size at risk for 25(OH)D deficiency (<30 ng/mL) (Sample sizes included in each covariate description is not identical to the total sample due to missing values)

**BMI: Body mass index (Kg/m^2^);

Considering each sleep parameter separately as an exposure variable in simple logistic regression models, moderate and severe OSA, objective short sleep duration and AI (3^th^ and 4^th^ quartiles in comparison to the lowest quartile) were associated with the risk of 25(OH) deficiency (moderate OSA: OR for 25(OH)D<30 = 1.90, 95% CI: 1.21–2.98, p<0.01; severe OSA: OR for 25(OH)D<30 = 1.64, 95% CI: 1.06–2.54, p = 0.03; short sleep duration: OR for 25(OH)D<30 = 1.49, 95% CI: 1.09–2.04, p = 0.01; AI (quartile 3): OR for 25(OH)D<30 = 1.64, 95%CI: 1.04–2.68, p = 0.03; AI (quartile 4): OR for 25(OH)D<30 = 1.69, 95%CI: 1.09–2.62, p = 0.02) (Table 2). During the final modeling, collinearity was detected due to the inclusion of arousal index. Therefore, the final multiple logistic regression model, keeping the adjustment for age, gender, race/ethnicity, obesity, smoking, hypertension, diabetes, sedentarism, seasonality and creatinine serum levels, included OSA and objective short sleep duration. In the final analysis, only OSA and objective short sleep duration still showed significant associations with the risk of 25(OH) deficiency (moderate OSA: OR for 25(OH)D<30 = 2.21, 95% CI: 1.35–3.64, p<0.01; severe OSA: OR for 25(OH)D<30 = 1.78, 95% CI: 1.06–3.00, p = 0.03; short sleep duration: OR for 25(OH)D<30 = 1.61, 95% CI: 1.15–2.26, p = 0.01) (Table 3). The subgroup analysis stratified by age groups (<50 years and ≥50 years) is showed in S1 and S2 Tables. After conducting this additional secondary analysis, only the subgroup ≥50 years maintained the similar results observed in the total sample regarding the risk of 25(OH) deficiency (moderate OSA: OR for 25(OH)D<30 = 2.40, 95% CI: 1.26–4.54, p<0.01; severe OSA: OR for 25(OH)D<30 = 1.94, 95% CI: 0.97–3,86, p = 0.06; short sleep duration: OR for 25(OH)D<30 = 1.84, 95% CI: 1.17–2.79, p = 0.01).

**Table 2:** Simple logistic regression analysis of PSG parameters estimating unadjusted odds ratios for the risk of low serum 25(OH)D (<30 ng/mL compared to ≥30ng/mL).

		OR	95% CI	P value*
Sleep efficiency (< 80%)**		0.99	0.58–1.71	0.98
**Sleep latency (≥ 30min)**		0.80	0.56–1.15	0.30
**N1 (% TST)**				
***Quartile 1***	< 2.37	0.75	0.47–1.17	0.21
***Quartile 2***	2.37 to < 4.55	Reference		
***Quartile 3***	4.55 to < 7.08	0.85	0.55–1.34	0.49
***Quartile 4***	≥ 7.08	1.15	0.75–1.77	0.53
**N2 (% TST)**				
***Quartile 1***	< 51.0	1.30	0.82–2.05	0.26
***Quartile 2***	51.0 to < 57.6	Reference		
***Quartile 3***	57.6 to < 65.5	1.44	0.91–2.27	0.12
***Quartile 4***	≥ 65.5	1.47	0.95–2.28	0.09
**N3 (% TST)**				
***Quartile 1***	< 12.0	1.44	0.93–2.22	0.10
***Quartile 2***	12.0 to < 17.3	1.20	0.77–1.86	0.42
***Quartile 3***	17.3 to < 23.6	1.30	0.84–2.01	0.24
***Quartile 4***	≥ 23.6	Reference		
**REM (% TST)**				
***Quartile 1***	< 13.6	1.23	0.79–1.92	0.35
***Quartile 2***	13.6 to < 18.8	1.53	0.99–2.35	0.06
***Quartile 3***	18.8 to < 22.9	1.40	0.90–0.17	0.13
***Quartile 4***	≥ 22.9	Reference		
**Arousal index (number/h TST)**				
***Quartile 1***	< 3.36	Reference		
***Quartile 2***	3.36 to < 9.94	0.95	0.60–1.52	0.84
***Quartile 3***	9.94 to < 26.7	**1.64**	**1.04–2.58**	**0.03**
***Quartile 4***	≥ 26.7	**1.69**	**1.09–2.62**	**0.02**
**PLMI (number/h TST)**				
***Quartile 1***	< 4.3	Reference		
***Quartile 2***	4.3 to < 7.8	3.40	0.93–12.49	0.07
***Quartile 3***	7.8 to < 21.9	2.35	0.63–8.72	0.20
***Quartile 4***	≥ 21.9	2.34	0.85–6.43	0.10
**Objective short sleep duration (<6 h)**		**1.49**	**1.09–2.04**	0.01*
Obstructive sleep apnea***	***Mild***	1.15	0.75–1.75	0.52
	***Moderate***	**1.90**	**1.21–2.98**	<0.01*
	***Severe***	**1.64**	**1.06–2.54**	0.03*

* *P*-values<0.05 were considered significant

** Sleep Efficiency: Total Sleep Time (TST)/Time in Bed x 100

*** No-OSA status was considered as reference

CI: confidence intervals; REM: rapid eye movement; PLMI: periodic limb movement index; TST: total sleep time

**Table 3:** Multiple logistic regression analysis* estimating adjusted odds ratios for the risk of low serum 25(OH)D (<30 ng/mL compared to ≥30 ng/mL).

	aOR	95% CI	*P*-value**
**Age**	0.98	0.96–1.00	0.06**
**Gender (Female)**	0.83	0.58–1.19	0.31
**Race/Ethnicity (African American)**	1.02	0.59–1.74	0.95
**Sedentarism**	0.88	0.62–1.25	0.48
**Current Smoking**	0.70	0.42–1.13	0.14
**Hypertension**	1.10	0.75–1.60	0.64
**Diabetes**	1.42	0.82–2.46	0.21
**Obesity (BMI ≥30)**	0.81	0.55–1.19	0.28
**Seasonality (winter)**	0.88	0.59–1.29	0.51
**Serum creatinine**	0.96	0.75–1.23	0.74
**Objective short sleep duration (< 6h)**	**1.61**	**1.15–2.26**	0.01**
Obstructive sleep apnea categories***			
***Mild***	1.11	0.70–1.75	0.65
***Moderate***	**2.21**	**1.35–3.64**	<0.01**
***Severe***	**1.78**	**1.06–3.00**	0.03**

* Sample size (n = 624) included in the final analysis was not identical to the total sample due to missing values on covariates

** *P*-values<0.05 were considered significant

*** No OSA status was considered as reference

aOR: adjusted odds ratios; CI: confidence intervals; BMI: Body mass index (Kg/m^2^)

## Discussion

Recently, some studies have suggested that sleep disorders are associated with decreased levels of serum 25(OH)D in different populations [10], [15]–[18]. Our study confirmed this general hypothesis by showing that moderate to severe OSA and PSG-measured short sleep duration are independently associated with the risk of serum 25(OH)D deficiency in a sample including predominantly middle-aged and older participants, after controlling for major potential confounders. A subgroup analysis also showed that these associations persisted only in the participants who were ≥50 years.

Short sleep duration, as well as poor sleep quality and efficiency, have been found more frequently in individuals with lower serum 25(OH)D levels compared to those with sufficient levels. [15], [16] Several factors may explain this association, among which a hypothetical review has suggested that lower 25(OH)D levels might lead to sleep impairment and daytime sleepiness through the increase of proinflammatory mediators, such as tumor necrosis factor alpha and interleukin-1. [6] On the other hand, short sleep duration could also favor 25(OH)D deficiency as insomnia and insufficient sleep are linked to changes in dietary habits and outdoor activity’s patterns. [26] In fact, as Afro-descendants are more susceptible to 25(OH)D deficiency as well as higher indexes of daytime sleepiness, a possible link between these two factors may exist. [27]

Concurrently, OSA has also been related to higher pro-inflammatory markers commonly associated with 25(OH)D deficiency,[28] which in turn may increase OSA risk by predisposing individuals to myopathy, tonsillar hypertrophy, and rhinitis, and thus leading to higher chance of upper airway collapse during sleep. [6] Otherwise, the recently reported effects of CPAP on 25(OH)D levels in patients with OSA supports the hypothesis that OSA can interfere with the metabolic pathways related to the 25(OH)D synthesis. [13], [14] As proposed in patients with chronic obstructive pulmonary diseases,[29] OSA-related hypoxia is putatively linked to reduced levels of serum 25(OH)D. Therefore, intermittent hypoxemia during sleep may explain at least in part the mechanisms involved in the relationship between OSA and 25(OH)D deficiency. [30] Furthermore, 25(OH)D pleiotropic effects may be associated with an extended range of associations between sleep and health outcomes including mortality. [1] Sleep curtailment, as well as OSA, have been consistently related to common chronic conditions, such as hypertension, diabetes, obesity and cardiovascular diseases. [31], [32] Similarly, 25(OH)D deficiency has also been linked to the development of these comorbidities. [1] Therefore, a possible explanation for the existing association of sleep-related parameters or disorders with cardiometabolic outcomes could be due partially to 25(OH)D deficiency.

Interestingly, after stratifying the analysis according to age range, the associations observed in the total sample were only detected in the subgroup with ≥50 years. Although this result derives from a secondary analysis, the increasing relevance of both vitamin D deficiency and sleep disorders in the older population may explain this finding. Indeed, epidemiological studies have found a higher prevalence of vitamin D deficiency among older adults. [33], [34] Additionally, evidence suggests an age-related decline in VDR expression and 1,25-dihydroxycholecalciferol (1,25(OH)D) activity in some body tissues. [35] The impact of this physiological change in the brain needs further investigation. On the other hand, sleep disorders and circadian rhythm deregulation are also more common among older people compared to adults. [36] Aging is one of the main risk factors for OSA, and sleep curtailment is more observed in the elderly. [24], [37] Therefore, OSA and shorter sleep duration in older adults may explain, at least in part, the age-changes in vitamin D levels and metabolism. Furthermore, another possible explanation for the increasing prevalence of sleep disorders in older adults can derive from studies targeting the vitamin D metabolism. However, new studies are necessary to test theses hypotheses.

This is an observational study that cross-sectionally evaluated the association between objectively measured sleep parameters, demographic, and clinical factors with serum 25(OH)D levels. By using a relatively large sample size and controlling the analyses for the most common and important potential confounders in the associations between the risk of 25(OH)D deficiency and PSG-derived sleep parameters, we were able to efficiently and precisely measure the independent associations between the studied factors. However, our conclusions have some limitations. The study design does not allow any inference about causality between lower 25(OH)D and sleep parameters. Additionally, other potential confounders in the studied associations could not be measured and included in the analyses. As an example, although we included indirect measurements of sunlight exposure, such as seasonality and inactive lifestyle status, more reliable methods of outdoor activities and sunlight exposition could not be used. Other factors commonly related to 25(OH)D deficiency and sleep disorders, such as depression, pain and 25(OH)D dietary intake, were not assessed in this study. [9] Moreover, the current lack of a consensus regarding the appropriate cut-off levels for 25(OH)D deficiency led us to consider the US Endocrine Society Clinical Practice Guideline definition for vitamin D insufficiency (25(OH)D<30 ng/mL) in our study. [23] In fact, it has been suggested that individuals with 25(OH)D<30 ng/mL are at risk for vitamin D deficiency or in suboptimal vitamin D levels. However, more studies are expected to define the adequate vitamin D levels related to the reduction of several clinical outcomes, including those directly or indirectly associated to sleep disorders.

Despite the fact that PSGs were performed respecting the habitual bedtime of the participants, data interpretation was limited by the single night PSG recordings and the consequent problems derived from potential poor compliance or lack of habituation during the sleep study. Although we have performed a statistical analysis looking at the effects of sleep efficiency on the risk of vitamin D deficiency to reduce the source of bias related to a single night measurement effect, little is known about the presence of participants with a specific short sleep duration phenotype in our sample, which might aid to define the underlying mechanisms involved in the association between 25(OH)D levels and short sleep duration. Nevertheless, short sleep duration, regardless of its cause, seems to be an independent risk factor for main clinical outcomes, including obesity, type 2 diabetes, hypertension, and cardiovascular disease. [38]

## Conclusions

To our knowledge, this is the first study to demonstrate that moderate to severe OSA and objective short sleep duration are independently associated with the risk of 25(OH)D deficiency in an adult sample population. An age-related mechanism related to vitamin D serum levels and metabolism, as well as the higher prevalence of sleep complains and disorders in older adults, may support our findings. As both OSA and sleep curtailment as well as 25(OH)D deficiency are increasingly frequent and found to be linked to some of the most prevalent chronic diseases worldwide, implementing strategies to reduce the impact of these conditions on morbimortality are welcome. Therefore, future studies, mainly clinical trials, testing the effects of sleep-related interventions on serum 25(OH)D status or vice-versa are expected.

## Supporting information

S1 Table Multiple logistic regression analysis* estimating adjusted odds ratios for the risk of serum 25(OH)D deficiency (<30 ng/mL compared to ≥30ng/mL) in participants <50 years. (DOCX)

S2 Table Multiple logistic regression analysis estimating adjusted odds ratios for the risk of serum 25(OH)D deficiency (<30 ng/mL compared to ≥30ng/mL) in participants ≥50 years. (DOCX)

S1 File ERA project survey and questionnaires applied for the assessment of socio-demographic and clinical characteristics of the participants. [ERA ID participant.doc].
(DOC)

S2 File ERA project baseline dataset. [Dataset.xls].
(XLS)

## References

[1] Pludowski P, Holick MF, Pilz S, Wagner CL, Hollis BW, Grant WB, et al Vitamin D effects on musculoskeletal health, immunity, autoimmunity, cardiovascular disease, cancer, fertility, pregnancy, dementia and mortality—A review of recent evidence. Autoimmun Rev. 2013 8;12(10):976–89. 23542507 10.1016/j.autrev.2013.02.004

[2] Garcion E, Wion-Barbot N, Montero-Menei CN, Berger F, Wion D. New clues about vitamin D functions in the nervous system. Trends Endocrinol Metab TEM. 2002 4;13(3):100–5. 11893522 10.1016/s1043-2760(01)00547-1

[3] Eyles DW, Smith S, Kinobe R, Hewison M, McGrath JJ. Distribution of the Vitamin D receptor and 1α-hydroxylase in human brain. J Chem Neuroanat. 2005 1;29(1):21–30. doi: 10.1016/j.jchemneu.2004.08.006 15589699 10.1016/j.jchemneu.2004.08.006

[4] Saper CB, Scammell TE, Lu J. Hypothalamic regulation of sleep and circadian rhythms. Nature. 2005 10 27;437(7063):1257–63. doi: 10.1038/nature04284 16251950 10.1038/nature04284

[5] Andersen ML, Tufik S. Vitamin D as an Underlying Factor in Sleep-Related Issues. J Clin Sleep Med [Internet]. 2012 12 15 [cited 2016 Dec 30]; http://www.aasmnet.org/jcsm/ViewAbstract.aspx?pid=28732 10.5664/jcsm.2268 PMC3501667 23243404

[6] McCarty DE, Chesson AL, Jain SK, Marino AA. The link between vitamin D metabolism and sleep medicine. Sleep Med Rev. 2014 8;18(4):311–9. doi: 10.1016/j.smrv.2013.07.001 24075129 10.1016/j.smrv.2013.07.001

[7] Puckrin R, Iqbal S, Zidulka A, Vasilevsky M, Barre P. Renoprotective effects of continuous positive airway pressure in chronic kidney disease patients with sleep apnea. Int Urol Nephrol. 2015 11;47(11):1839–45. doi: 10.1007/s11255-015-1113-y 26424500 10.1007/s11255-015-1113-y

[8] Nakashima A, Yokoyama K, Yokoo T, Urashima M. Role of vitamin D in diabetes mellitus and chronic kidney disease. World J Diabetes. 2016;7(5):89 doi: 10.4239/wjd.v7.i5.89 26981182 10.4239/wjd.v7.i5.89 PMC4781904

[9] Evatt ML. Vitamin D Associations and Sleep Physiology—Promising Rays of Information. SLEEP [Internet]. 2015 2 1 [cited 2016 Oct 21]; http://www.journalsleep.org/ViewAbstract.aspx?pid=29854 10.5665/sleep.4386 PMC4288595 25581928

[10] Bozkurt NC, Cakal E, Sahin M, Ozkaya EC, Firat H, Delibasi T. The relation of serum 25-hydroxyvitamin-D levels with severity of obstructive sleep apnea and glucose metabolism abnormalities. Endocrine. 2012 6;41(3):518–25. doi: 10.1007/s12020-012-9595-1 22246808 10.1007/s12020-012-9595-1

[11] Upala S, Sanguankeo A. Association between 25-Hydroxyvitamin D and Obstructive Sleep Apnea: A Systematic Review and Meta-Analysis. J Clin Sleep Med. 2015 11 15;11(11):1347–1347. doi: 10.5664/jcsm.5208 26235155 10.5664/jcsm.5208 PMC4623137

[12] Kerley CP, Hutchinson K, Bolger K, McGowan A, Faul J, Cormican L. Serum Vitamin D Is Significantly Inversely Associated with Disease Severity in Caucasian Adults with Obstructive Sleep Apnea Syndrome. SLEEP. 2016 2 1;39(02):293–300. 26414899 10.5665/sleep.5430 PMC4712400

[13] Liguori C, Romigi A, Izzi F, Mercuri NB, Cordella A, Tarquini E, et al Continuous Positive Airway Pressure Treatment Increases Serum Vitamin D Levels in Male Patients with Obstructive Sleep Apnea. J Clin Sleep Med [Internet]. 2015 6 15 [cited 2016 Oct 21]; http://www.aasmnet.org/jcsm/ViewAbstract.aspx?pid=30052 10.5664/jcsm.4766 PMC4442220 25766695

[14] Liguori C, Izzi F, Mercuri NB, Romigi A, Cordella A, Tarantino U, et al Vitamin D status of male OSAS patients improved after long-term CPAP treatment mainly in obese subjects. Sleep Med. 2017 1;29:81–5. doi: 10.1016/j.sleep.2016.08.022 27964863 10.1016/j.sleep.2016.08.022

[15] Kim JH, Chang JH, Kim DY, Kang JW. Association Between Self-Reported Sleep Duration and Serum Vitamin D Level in Elderly Korean Adults. J Am Geriatr Soc. 2014 12;62(12):2327–32. doi: 10.1111/jgs.13148 25516029 10.1111/jgs.13148

[16] Pande RU, Chandrasekhar R, Kaplish N, Rifkin DI. Low serum vitamin D concentration as a predictor of short sleep duration: a NHANES 2005–2006 analysis. 32(Abstract Suppl). 2009;A136.

[17] Massa J, Stone KL, Wei EK, Harrison SL, Barrett-Connor E, Lane NE, et al Vitamin D and Actigraphic Sleep Outcomes in Older Community-Dwelling Men: The MrOS Sleep Study. SLEEP [Internet]. 2015 2 1 [cited 2016 Oct 21]; http://www.journalsleep.org/ViewAbstract.aspx?pid=29865 10.5665/sleep.4408 PMC4288606 25581929

[18] Bertisch SM, Sillau S, de Boer IH, Szklo M, Redline S. 25-Hydroxyvitamin D Concentration and Sleep Duration and Continuity: Multi-Ethnic Study of Atherosclerosis. SLEEP [Internet]. 2015 8 1 [cited 2016 Oct 21]; http://www.journalsleep.org/ViewAbstract.aspx?pid=30126 10.5665/sleep.4914 PMC4507736 25669179

[19] Rechtschaffen Allan, Kales Anthony. A manual of standardized terminology, techniques and scoring system of sleep stages in human subjects. Los Angeles: University of California Los Angeles Brain Information Service; 1968. (National Institutes of Health publication).

[20] Iber C, Ancoli-Israel S, Chesson A, Quan S. The AASM manual for the scoring of sleep and associated events: rules, terminology, and technical specifications. American Academy of Sleep Medicine; 2007.

[21] Koivula M-K, Matinlassi N, Laitinen P, Risteli J. Four automated 25-OH total vitamin D immunoassays and commercial liquid chromatography tandem-mass spectrometry in Finnish population. Clin Lab. 2013;59(3–4):397–405. 23724631 10.7754/clin.lab.2012.120527

[22] Ross AC. The 2011 report on dietary reference intakes for calcium and vitamin D. Public Health Nutr. 2011 5;14(05):938–9. 21492489 10.1017/S1368980011000565

[23] Holick MF, Binkley NC, Bischoff-Ferrari HA, Gordon CM, Hanley DA, Heaney RP, et al Evaluation, Treatment, and Prevention of Vitamin D Deficiency: an Endocrine Society Clinical Practice Guideline. J Clin Endocrinol Metab. 2011 7;96(7):1911–30. doi: 10.1210/jc.2011-0385 21646368 10.1210/jc.2011-0385

[24] Kleinbaum DG, Klein M. Logistic regression, a self-learning text. Third Edtion New York: Springer; 2010.

[25] Brouwer-Brolsma EM, Vaes AMM, van der Zwaluw NL, van Wijngaarden JP, Swart KMA, Ham AC, et al Relative importance of summer sun exposure, vitamin D intake, and genes to vitamin D status in Dutch older adults: The B-PROOF study. J Steroid Biochem Mol Biol [Internet]. 2015 8 [cited 2016 Oct 21]; Available from: http://linkinghub.elsevier.com/retrieve/pii/S0960076015300455 10.1016/j.jsbmb.2015.08.008 26275945

[26] de Oliveira DL, Hirotsu C, Tufik S, Andersen ML. Vitamin D and Sleep Apnea: Beyond a Simple Association. J Clin Sleep Med. 2015 11 15;11(11):1345–1345. doi: 10.5664/jcsm.5206 26350605 10.5664/jcsm.5206 PMC4623136

[27] Unnikrishnan D, Jun J, Polotsky V. Inflammation in sleep apnea: An update. Rev Endocr Metab Disord. 2015 3;16(1):25–34. doi: 10.1007/s11154-014-9304-x 25502450 10.1007/s11154-014-9304-x PMC4346503

[28] Persson LJP, Aanerud M, Hiemstra PS, Hardie JA, Bakke PS, Eagan TML. Chronic Obstructive Pulmonary Disease Is Associated with Low Levels of Vitamin D. Hartl D, editor. PLoS ONE. 2012 6 21;7(6):e38934 doi: 10.1371/journal.pone.0038934 22737223 10.1371/journal.pone.0038934 PMC3380863

[29] Ahmad Y, Sharma NK, Garg I, Ahmad MF, Sharma M, Bhargava K. An Insight into the Changes in Human Plasma Proteome on Adaptation to Hypobaric Hypoxia. Khan RH, editor. PLoS ONE. 2013 7 2;8(7):e67548 doi: 10.1371/journal.pone.0067548 23844025 10.1371/journal.pone.0067548 PMC3699623

[30] Itani O, Jike M, Watanabe N, Kaneita Y. Short sleep duration and health outcomes: a systematic review, meta-analysis, and meta-regression. Sleep Med [Internet]. 2016 8 [cited 2016 Oct 21]; http://linkinghub.elsevier.com/retrieve/pii/S1389945716301381 10.1016/j.sleep.2016.08.006 27743803

[31] Bauters F, Rietzschel ER, Hertegonne KBC, Chirinos JA. The Link Between Obstructive Sleep Apnea and Cardiovascular Disease. Curr Atheroscler Rep [Internet]. 2016 1 [cited 2016 Oct 21];18(1). Available from: http://link.springer.com/10.1007/s11883-015-0556-z 10.1007/s11883-015-0556-z 26710793

[32] Ardawi M-SM, Sibiany AM, Bakhsh TM, Qari MH, Maimani AA. High prevalence of vitamin D deficiency among healthy Saudi Arabian men: relationship to bone mineral density, parathyroid hormone, bone turnover markers, and lifestyle factors. Osteoporos Int J Establ Result Coop Eur Found Osteoporos Natl Osteoporos Found USA. 2012 2;23(2):675–86. 10.1007/s00198-011-1606-1 21625888

[33] Zhen D, Liu L, Guan C, Zhao N, Tang X. High prevalence of vitamin D deficiency among middle-aged and elderly individuals in northwestern China: its relationship to osteoporosis and lifestyle factors. Bone. 2015 2;71:1–6. doi: 10.1016/j.bone.2014.09.024 25284157 10.1016/j.bone.2014.09.024

[34] Sanders KM, Scott D, Ebeling PR. Vitamin D deficiency and its role in muscle-bone interactions in the elderly. Curr Osteoporos Rep. 2014 3;12(1):74–81. doi: 10.1007/s11914-014-0193-4 24488588 10.1007/s11914-014-0193-4

[35] Piovezan RD, Abucham J, Dos Santos RVT, Mello MT, Tufik S, Poyares D. The impact of sleep on age-related sarcopenia: Possible connections and clinical implications. Ageing Res Rev. 2015 9;23(Pt B):210–20. doi: 10.1016/j.arr.2015.07.003 26216211 10.1016/j.arr.2015.07.003

[36] Epstein LJ, Kristo D, Strollo PJ, Friedman N, Malhotra A, Patil SP, et al Clinical guideline for the evaluation, management and long-term care of obstructive sleep apnea in adults. J Clin Sleep Med JCSM Off Publ Am Acad Sleep Med. 2009 6 15;5(3):263–76. PMC2699173 19960649

[37] Moraes W, Piovezan R, Poyares D, Bittencourt LR, Santos-Silva R, Tufik S. Effects of aging on sleep structure throughout adulthood: a population-based study. Sleep Med. 2014 4;15(4):401–9. doi: 10.1016/j.sleep.2013.11.791 24657204 10.1016/j.sleep.2013.11.791

[38] Dashti HS, Scheer FA, Jacques PF, Lamon-Fava S, Ordovás JM. Short sleep duration and dietary intake: epidemiologic evidence, mechanisms, and health implications. Adv Nutr Bethesda Md. 2015 11;6(6):648–59. 10.3945/an.115.008623 PMC4642416 26567190

