# Supplementary material for: Obstructive sleep apnea and objective short sleep duration are independently associated with the risk of serum vitamin D deficiency
Source: PLoS One. 2017 Jul 7;12(7):e0180901. doi: 10.1371/journal.pone.0180901 (PMC5501615; doi:10.1371/journal.pone.0180901)
Supplement: S1 Table — (DOCX) [file pone.0180901.s001.docx]

**Table S1.** Multiple logistic regression analysis* estimating adjusted odds ratios for the risk of serum 25(OH)D deficiency (<30 ng/mL compared to ≥30ng/mL) in participants <50 years.

|  | aOR | 95% CI | | *P*-value* |
| --- | --- | --- | --- | --- |
| Age | 1.01 | 0.95-1.07 | | 0.74 |
| Gender (Female) | 0.61 | 0.29-1.25 | | 0.18 |
| Race/Ethnicity (African American) | 0.40 | 0.31-1.60 | | 0.95 |
| Sedentarism | 0.95 | 0.54-1.70 | | 0.88 |
| Current smoking | 0.70 | 0.60-2.45 | | 0.59 |
| Hypertension | 1.18 | 0.57-2.41 | | 0.66 |
| Diabetes | 1.00 | 0.32-3.13 | | 0.99 |
| Obesity (BMI ≥30) | 0.83 | 0.43-1.60 | | 0.28 |
| Seasonality (winter) | 0.92 | 0.46-1.84 | | 0.82 |
| Serum creatinine | 0.95 | 0.07-4.87 | | 0.62 |
| Objective short sleep duration (<6 h) | 1.48 | 0.86-2.54 | | 0.16 |
| Obstructive sleep apnea categories** |  |  |  | |
| *Mild* | 1.10 | 0.55-2.22 | | 0.29 |
| *Moderate* | 1.82 | 0.78-4.25 | | 0.17 |
| *Severe* | 1.58 | 0.67-3.72 | | 0.29 |

* *P*-values<0.05 were considered significant

** No OSA status was considered as reference

aOR: adjusted odds ratios; CI: confidence intervals, BMI: Body mass index (Kg/m^2^)
