# Supplementary material for: Obstructive sleep apnea and objective short sleep duration are independently associated with the risk of serum vitamin D deficiency
Source: PLoS One. 2017 Jul 7;12(7):e0180901. doi: 10.1371/journal.pone.0180901 (PMC5501615; doi:10.1371/journal.pone.0180901)
Supplement: S2 Table — (DOCX) [file pone.0180901.s002.docx]

**Table S2.** Multiple logistic regression analysis estimating adjusted odds ratios for the risk of serum 25(OH)D deficiency (<30 ng/mL compared to ≥30ng/mL) in participants ≥50 years.

|  | aOR | 95% CI | *P*-value* |
| --- | --- | --- | --- |
| Age | **0.98** | **0.91-0.99** | **0.01**** |
| Gender (Female) | 0.85 | 0.52-1.38 | 0.50 |
| Race/Ethnicity (African American) | 1.34 | 0.64-2.81 | 0.44 |
| Sedentarism | 0.79 | 0.50-1.24 | 0.30 |
| Current Smoking | **0.37** | **0.17-0.80** | **0.01**** |
| Hypertension | 1.14 | 0.72-1.82 | 0.58 |
| Diabetes | 1.55 | 0.81-2.97 | 0.18 |
| Obesity (BMI ≥30) | 0.75 | 0.45-1.23 | 0.25 |
| Seasonality (winter) | 0.86 | 0.53-1.40 | 0.54 |
| Serum creatinine | 0.96 | 0.79-1.18 | 0.72 |
| Objective short sleep duration (< 6 h) | **1.84** | **1.17-2.89** | **0.01**** |
| Obstructive sleep apnea categories** |  |  |  |
| *Mild* | 1.11 | 0.59-2.08 | 0.75 |
| *Moderate* | **2.40** | **1.26-4.54** | **<0.01**** |
| *Severe* | 1.94 | 0.97-3.86 | 0.06 |

* *P*-values<0.05 were considered significant

** No OSA status was considered as reference

ORa: adjusted odds ratios; CI: confidence intervals; BMI: Body mass index (Kg/m^2^)
