# Supplementary material for: Obstructive sleep apnea and objective short sleep duration are independently associated with the risk of serum vitamin D deficiency
Source: PLoS One. 2017 Jul 7;12(7):e0180901. doi: 10.1371/journal.pone.0180901 (PMC5501615; doi:10.1371/journal.pone.0180901)

| ***Participant´s identification file*** | | | |
| --- | --- | --- | --- |
| LAST NAME, FIRST NAME | | | |
|  | | | |
| OCCUPATION | | | |
| Name and Address of Employer | | | From |
|  | | |  |
|  | | | |
| **EDUCATION** |  | | **GENDER** |
|  |  | | ( ) M  ( ) F |
|  | | | |
| **ADDRESS** | | |  |
|  | | |  |
| **ID NUMBER ISSUE DATE STATE** | | | |
|  |  | |  |
|  |  | |  |
| **SOCIAL SECURITY NUMBER (CPF)** | | | |
| PHONE NUMBER TO BE CONTACTED | | | |
| **BIRTH DATE** | | | |
| **Public Health System - Health card number (if applied)** | | | |
| **Medical Insurance (Company and number, if applied)** | | | |
| **Signature:** | | **Date: xx/xx/xxxx** | |
|  | |  | |

| ***Ficha de Identificação do Participante*** | | | |
| --- | --- | --- | --- |
| ULTIMO NOME, PRIMEIRO NOME | | | |
|  | | | |
| OCUPAÇÃO | | | |
| Nome e endereço do local de trabalho | | | Desde quando |
|  | | |  |
|  | | | |
| **EDUCAÇÃO** |  | | **Gênero** |
|  |  | | ( ) M  ( ) F |
|  | | | |
| **ENDEREÇO** | | |  |
|  | | |  |
| **NÚMERO ID DATA ESTADO** | | | |
|  |  | |  |
|  |  | |  |
| **CPF** | | | |
| TELEFONE DE CONTATO | | | |
| **NASCIMENTO** | | | |
| **Cartão do SUS** | | | |
| **Seguro de saúde** | | | |
| **Assinatura:** | | **Data: xx/xx/xxxx** | |
|  | |  | |

**Cardiovascular Risk Stratification in Obstructive Sleep Apnea – ERA STUDY**

1. Do you have arterial hypertension? Yes ____ No_____
2. What is your systolic arterial pressure? ________mmHg
3. What is your diastolic arterial pressure? ________mmHg
4. Do you have diabetes? Yes ____ No_____
5. Do you use insulin? Yes ____ No_____
6. Are you a smoker? Yes ____ No_____
7. Are you sedentary (defined as lack of physical activity in the last 6 months)?

Yes ____ No_____

1. Number of sleep hours

< 5___, 5:01-06:00___, 06:01-7:00___, 07:01-08:00___, 08:01-9:00___, > 09:00__

1. What´s the reason for sleeping less than 5 hours?

presence of a disease that disrupt sleep___ work___ child or relative´s care___

it´s enough sleep___ insomnia___

1. What´s the reason for sleeping more than 9 hours?

Disease___ it´s enough sleep___ others____

1. Do you have any family history of coronary disease?

Yes ____ No_____

1. Do you have any family history of sudden cardiac death?

Yes ____ No_____

1. Do you have any family history of hormonal replacement?

Yes ____ No_____

1. Do you have any family history alcohol or other drugs´ use?

Yes ____ No_____

1. Do you have any personal history of Chagas disease?

Yes ____ No_____

1. Do you have any personal history of chronic obstructive pulmonary disease?

Yes ____ No_____

1. Medications and dosages


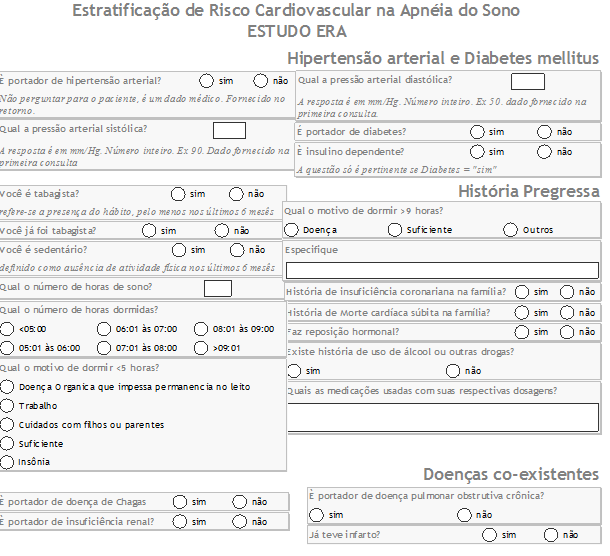

Supplement: S1 File — [ERA ID participant.doc]. (DOC) [file pone.0180901.s003.doc]
